# The Burden of Trachea, Bronchus, and Lung Cancer Attributable to Occupational Exposure From 1990 to 2019

**DOI:** 10.3389/fpubh.2022.928937

**Published:** 2022-06-17

**Authors:** Haifeng Li, Jingwen Guo, Hongsen Liang, Ting Zhang, Jinyu Zhang, Li Wei, Donglei Shi, Junhang Zhang, Zhaojun Wang

**Affiliations:** ^1^Department of Anesthesiology, Guangdong General Hospital, Guangzhou, China; ^2^Department of Thoracic Surgery, The Seventh Affiliated Hospital, Sun Yat-sen University, Shenzhen, China

**Keywords:** trachea, bronchus, lung cancer, occupational carcinogens, Global Burden of Disease, mortality, disability-adjusted life years (DALYs)

## Abstract

**Objectives:**

Occupational exposure to carcinogens is associated with trachea, bronchus, and lung (TBL) cancer. The objective of this study was to provide global and regional estimates of the burden of TBL cancer associated with occupational carcinogens (OCs) between 1990 and 2019.

**Methods:**

Age-standardized mortality rates (ASMR) and age-standardized disability-adjusted life years (DALYs) rates (ASDR) of TBL cancer related to exposure to OCs at the global and regional levels were extracted for 1990–2019 from the Global Burden of Disease 2019. Joinpoint regression was used to analyze trends in the ASMR and ASDR of TBL cancer burden related to OCs, and the annual percent change and the average annual percent change (AAPC) were recorded.

**Results:**

The mortality from TBL cancer related to exposure to OCs increased globally. The ASMR and ASDR decreased in both sexes and in men between 1990 and 2019. The AAPC of ASMR and ASDR decreased in men between 1990 and 2019, but increased in women. Asbestos accounted for the highest death number and beryllium accounted for the lowest; diesel engine exhaust caused the largest percentage change in death number (145.3%), in ASDR (14.9%), and in all ages DALY rates (57.6%). Asbestos accounted for the largest death number in high social development index (SDI) countries, whereas low-middle SDI countries had the largest percent change (321.4%). Asbestos was associated with decreased ASDR in high SDI countries and increased ASDR in low-middle SDI countries, and similar changes were observed for other OCs.

**Conclusions:**

The overall mortality and DALYs of TBL cancer burden related to OCs showed a decreasing trend between 1990 and 2019, whereas death number increased. Asbestos accounted for the highest death number. TBL cancer burden related to OCs decreased to different degrees in high, low, low-middle, and middle SDI countries, which showed variable levels of TBL cancer burden related to exposure to OCs (except asbestos).

## Introduction

Trachea, bronchus, and lung (TBL) cancer is the leading cause of cancer fatalities and the second major cause of new cancer cases globally (1, 2). In 2019, 2.26 million new cases of TBL cancer were reported, with 2.04 million deaths and 45.9 million disability-adjusted life years (DALYs) due to TBL cancer worldwide (1). In terms of global burden, lung cancer always ranks first among malignant tumors (3). Examining the trends and burden of TBL cancer can shed light on policy decision-making related to occupational protection.

Many factors contribute to the occurrence of TBL cancer, including genetic and environmental factors (4). Environmental factors, including occupational carcinogens (OCs) and air pollution, are important risk factors for TBL cancer (1). The three countries with the highest OC-attributable cancer burden worldwide were India (0.37 million), the US (0.71 million), and China (1.47 million) (5). The fraction of lung cancer deaths caused by OCs is estimated at 4–24% percent worldwide (6). However, there are no specific studies investigating the TBL cancer burden associated with OCs.

In this study, we present the results of the Global Burden of Disease (GBD) 2019 and analyze the current trends in mortality and DALYs of TBL cancer burden attributable to OCs between 1990 and 2019 using Joinpoint. For each OC, the respective attributable TBL cancer burden varied widely among countries. To better understand the cancer burden in different geographic locations, we assessed the burden and variation trends of TBL cancer attributable to OCs in countries with different social development index (SDI) values. We hope that our findings can provide a reference for policy planning and contribute to raise awareness of TBL cancer related to OCs.

## Methods

### Data Collection

The number of deaths and age-standardized DALY rates from TBL cancer related to OCs were obtained from the GBD 2019 for global data and from countries with different SDI values between 1990 and 2019 (http://ghdx.healthdata.org/gbd-results-tool). Of 87 risk factors, nine OCs were analyzed including arsenic, asbestos, beryllium, cadmium, chromium, diesel engine exhaust (DEE), nickel, polycyclic aromatic hydrocarbons (PAHs), and silica (7).

### Descriptive Study

The death number, DALYs, age-standardized mortality rate (ASMR) and ASDR due to OC exposure were calculated. A series of descriptive studies were conducted to evaluate the global status and percent change in TBL cancer due to OC between 1990 and 2019. Prism software (GraphPad Prism 8, USA) were used for data presentation. Data were summarized using microsoft excel and R (version 4.1.2), and we used R software to calculate the percentage change from 1990 to 2019.

### The Burden of TBL Cancer Across Countries With Different SDI Values

The SDI is a composite indicator of the developmental status of countries and territories. It is the geometric mean of the indices of total fertility rates under the age of 25 (TFU25), mean education for those aged 15 years or older, and the lagging distributed income per capita. SDI scores range from 0 to 1, and a higher SDI score indicates a better demographic and socioeconomic development of a country. Each GBD location has a yearly SDI score, and they are divided into five levels (high SDI, high-middle SDI, middle SDI, low-middle SDI, and low SDI). In this study, the burden of TBL cancer was calculated for countries with different SDI values. We also analyzed the number of deaths, the number of DALYs, ASMR, and ASDR at the 21 GBD regions (Supplementary Table S1).

### Analysis of Trends

Joinpoint regression was used to analyze trends in the ASMR and ASDR of TBL cancer burden related to exposure to OCs. The joinpoint regression program describes trends by connecting several different line segments at “joinpoints” and identifying points where the linear slope of a trend changes in a statistically significant way over time. Each *p*-value is determined using Monte Carlo methods, and the asymptotic significance level is maintained by using a Bonferroni correction (8). The annual percentage change (APC) and the average annual percentage change (AAPC) were used to describe the annual change in TBL cancer mortality and DALY rates between 1990 and 2019, APC and corresponding 95% confidence interval (CI), AAPC and 95% CI were recorded. Joinpoint regression software developed by the National Cancer Institute (version 4.1.9) was used. *P* < 0.05 was considered statistically significant.

## Results

### Descriptive Analysis of Mortality and DALY Trends

**Figure 1:**
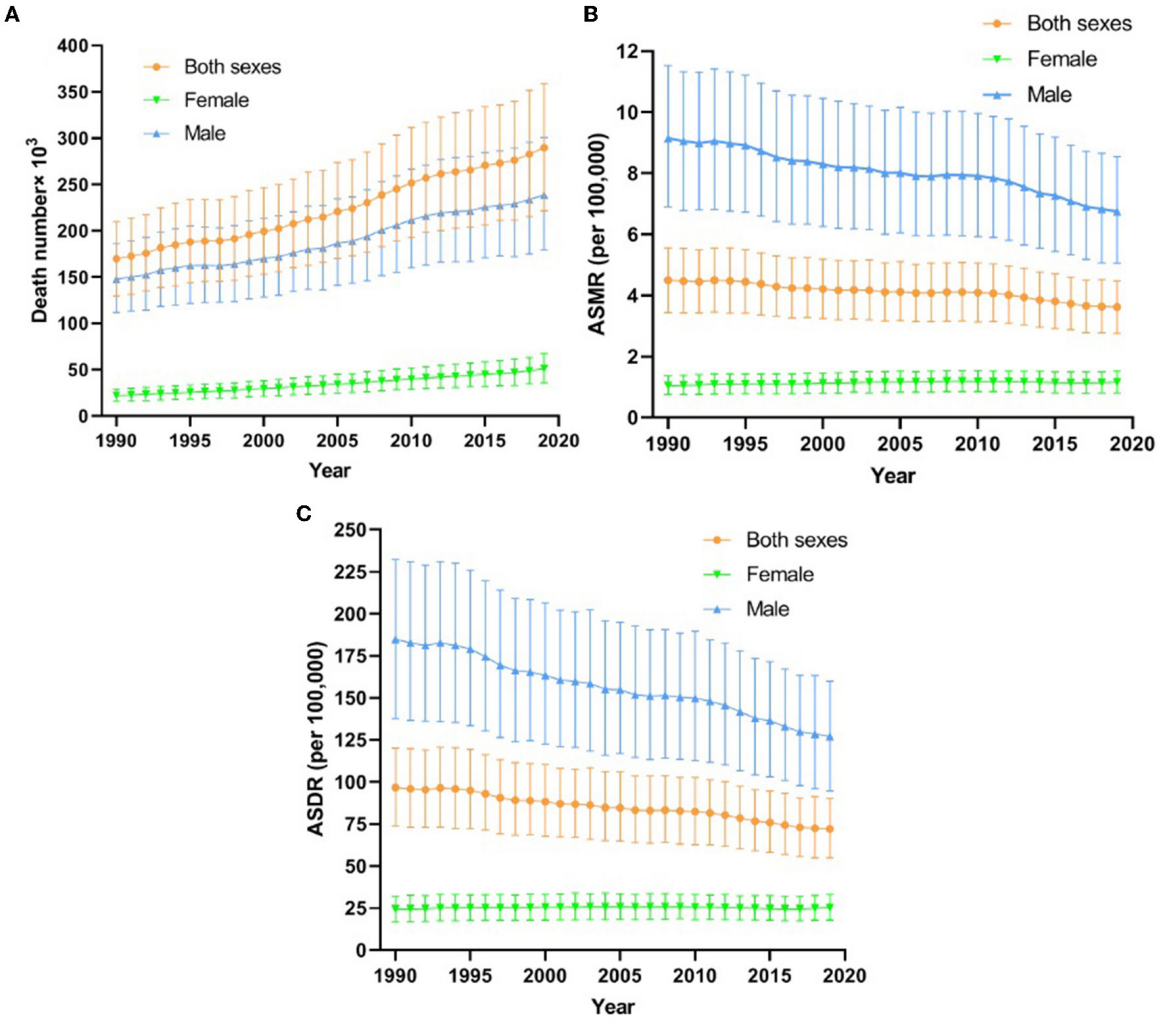
The deaths number **(A)**, ASMR **(B)** and ASDR **(C)** for TBL cancer from 1990 to 2019. DALY, disability adjusted life year; TBL cancer, tracheal, bronchus and lung cancer; ASMR, age-standardized mortality rates; ASDR, age-standardized DALY rates.

Figure 1 shows the death number, ASMR, and ASDR global trends for OC-attributable TBL cancer between 1990 and 2019. There were 289,793.1 (95% uncertainty interval (UI), 221524.1–359237.6) TBL cancer deaths attributable to OCs and the ASDR was 72.09 (95% UI, 54.87–90.39, per 100,000) in 2019 in both sexes; the majority of subjects included were men. Death number increased overall and slowly in women. ASMR and ASDR decreased gradually, and at a lower rate in women than in men (Figures 1B,C).

**Figure 2:**
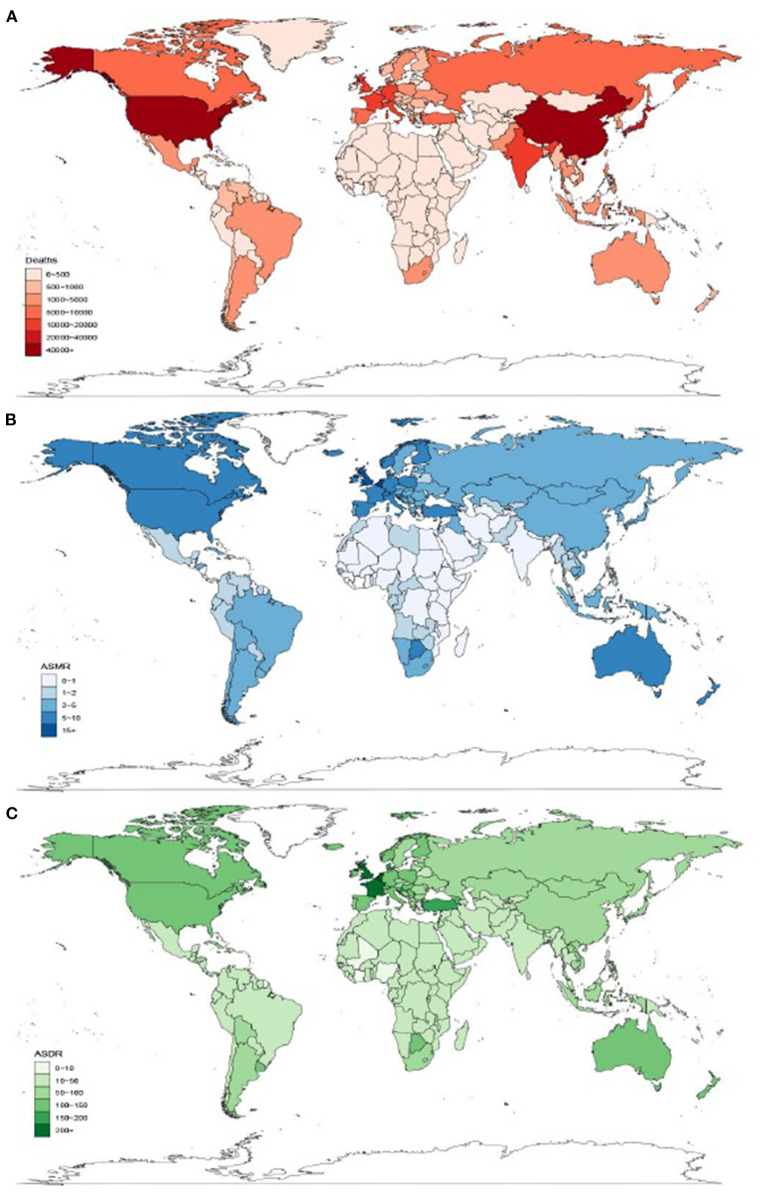
Global distribution of occupational carcinogen–attributable TBL cancer burden in terms of deaths **(A)**, ASMR **(B)**, and ASDR **(C)** in 2019. TBL cancer, tracheal, bronchus and lung cancer; ASMR, age-standardized mortality rate; ASDR, age-standardized DALY rate.

As shown in Figure 2A, China (62,861 deaths) and USA (41,075 deaths) were the two countries with the largest number of OC-attributable TBL cancer deaths in 2019. Monaco had the highest ASMR (16.7 per 100,000) in the world in 2019 (Figure 2B). Monaco (323.4 per 100,000) and Greenland (361 per 100,000) were the two countries with the greatest TBL cancer burden worldwide for ASDR in 2019 (Figure 2C).

Table 1 shows all age deaths, age-standardized DALY rates, and all ages DALYs of patients with TBL cancer attributable to OCs for both sexes between 1990 and 2019 globally. Among OCs, asbestos accounted for the highest death number and beryllium accounted for the lowest number of deaths. Asbestos-attributable TBL cancer deaths ranged from 122,205.8 (95% UI, 86779–158141.8) in 1990 to 198,703 (95% UI, 140365.9–257408) in 2019, indicating a change of 78.8%. DEE was associated with the largest percent change (145.3%) and arsenic the lowest percent change (62.6%) in death number. Arsenic, asbestos, nickel, and silica were the four OCs associated with a decrease in the change rate; DEE had the largest percent change (14.9%) in ASDR from 5.76 in 1990 to 6.62 in 2019. Arsenic was associated with a decrease in the percent change (−3.4%) and DEE had the largest percent change (57.6%) in all ages DALY rates from 4.62 in 1990 to 7.28 in 2019.

**Table 1:** Global all age deaths, age-standardized and all ages DALY of patients with TBL cancer attributable to 9 OCs for both sexes, 1990–2019.

**OC exposure**	**Deaths number** **(95% UI)**			**Age-standardized DALYs rates** **(95% UI, per 100,000 population)**			**All ages DALYs rates** **(95% UI, per 100,000 population)**		
	**1990**	**2019**	**Percentage change**	**1990**	**2019**	**Percentage change**	**1990**	**2019**	**Percentage change**
Arsenic	5458.1 (454.3–10416.4)	9760.2 (1554.1–17693.7)	62.6%	3.82 (0.37–7.26)	3.19 (0.53–5.72)	−33.4%	3.05 (0.3–5.79)	3.5 (0.58–6.28)	−3.4%
Asbesto	122205.8 (86779–158141.8)	198703 (140365.9–257408)	78.8%	62.6 (43.98–81.92)	41.71 (29.02–54.99)	−16.5%	45.05 (31.44–59.24)	43.5 (30.19–57.53)	14.8%
Beryllium	134.7 (107–163)	300.6 (243.9–367.2)	123.2%	0.1 (0.08–0.12)	0.1 (0.08–0.12)	0	0.08 (0.06–0.09)	0.11 (0.09–0.14)	37.5%
Cadmium	316.4 (258–378.9)	711.5 (582.7–853.9)	124.9%	0.23 (0.19–0.27)	0.24 (0.2–0.28)	4.3%	0.18 (0.15–0.22)	0.26 (0.22–0.31)	44.4%
Chromium	640.1 (559.2–728)	1499.4 (1286.7–1745.5)	134.2%	0.46 (0.4–0.52)	0.5 (0.43–0.58)	8.7%	0.37 (0.32–0.42)	0.55 (0.47–0.64)	48.6%
DEE	8036.3 (6835.8–9350.7)	19715.9 (16965.6–22916.5)	145.3%	5.76 (4.89–6.71)	6.62 (5.71–7.7)	14.9%	4.62 (3.92–5.39)	7.28 (6.27–8.47)	57.6%
Nickel	5248.1 (−330.2–15285.1)	9329.8 (535.7–24638.2)	77.8%	3.71 (−0.19–10.67)	3.07 (0.21–7.97)	−17.3%	2.96 (−0.15–8.5)	3.37 (0.24–8.75)	13.9%
PAHs	2251.5 (1872.3–2663.5)	5273.4 (4357.6–6241)	134.2%	1.61 (1.34–1.91)	1.77 (1.45–2.09)	9.9%	1.29 (1.07–1.53)	1.94 (1.6–2.29)	50.4%
Silica	30669.1 (12688–49130.5)	52984 (23792.1–84375.9)	72.8%	21.61 (8.99–34.57)	17.39 (7.84–27.66)	−19.5%	17.27 (7.19–27.6)	19.1 (8.6–30.37)	10.6%

*OCs, occupational carcinogens; DALYs, disability adjusted life years; DEE, diesel engine exhaust; PAHs, polycyclic aromatic hydrocarbons; TBL cancer, tracheal, bronchus and lung cancer; UI, uncertainty interval*.

### The Burden of TBL Cancer Across Countries With Different SDI Values

Table 2 presents all age deaths and age-standardized DALYs of patients with TBL cancer across countries with different SDI values. Asbestos accounted for the largest death number in high SDI countries, with a change from 87,162.1 (95% UI, 62583.3–110324.8) in 1990 to 119,560.5 (95% UI, 87245.8–151754.3) in 2019 (percent change, 37.2%), whereas low-middle SDI countries had the largest percentage change (321.4%). Middle SDI countries had the largest percentage change in death number related to exposure to arsenic (174.4%) and cadmium (175.4%). Low-middle SDI countries had the largest percentage change in death number related to exposure to beryllium (162.6%), chromium (194.2%), DEE (215.6%), nickel (158.6%), PAHs (192.9%), and silica (169.4%). Asbestos was related to a decrease in ASDR from 154.54 in 1990 to 96.1 in 2019, showing the largest percent change (−37.8%) in high SDI countries; however, in low-middle SDI countries, ASDR increased significantly (62.3%), and similar percent changes were observed for other OCs. Overall, there was decline to different degrees in high SDI countries in the burden of TBL cancer exposure to OCs, whereas low, low-middle, and middle SDI countries experienced varying levels of growth.

**Table 2:** All age deaths and age-standardized DALY of patients with TBL cancer exposure to OCs across countries with different SDI values.

**OC exposure**	**Location**	**Deaths number (95% UI)**			**ASDR (95% UI, per 100,000 population)**		
		**1990**	**2019**	**Percentage change**	**1990**	**2019**	**Percentage change**
Asbesto	High-middle SDI	26723.5 (18291.2–35936.9)	47642.2 (32335–64520.1)	78.3%	52.75 (35.81–71.25)	41.91 (27.66–57.59)	−20.5%
	High SDI	87162.1 (62583.3–110324.8)	119560.5 (87245.8–151754.3)	37.2%	154.54 (109.2–198.9)	96.1 (68.04–123.14)	−37.8%
	Low-middle SDI	1864.2 (1097.9–3331.5)	7856.3 (5005.6–11325.4)	321.4%	7.27 (4.27–13)	11.8 (7.47–17.2)	62.3%
	Low SDI	597.4 (256.4–1894)	1620.8 (825.9–4088.3)	171.3%	5.91 (2.57–18.87)	7.03 (3.58–18.04)	19%
	Middle SDI	5797.1 (3727.5–8500.6)	21930.4 (14176.5–31666.9)	278.3%	12.95 (8.29–18.92)	17.11 (10.99–24.77)	32.1%
Arsenic	High-middle SDI	1946.9 (200–3628.9)	3057.5 (581.8–5530.7)	57%	5.22 (0.55–9.71)	4.14 (0.81–7.48)	−20.7%
	High SDI	2011.3 (−422.4–4325.7)	2607.2 (−487.3– 5578.9)	29.6%	5.71 (−1.19–12.27)	3.95 (−0.7–8.43)	−30.8%
	Low-middle SDI	295.4 (107.7–483.1)	805.9 (298.1–1333.9)	172.8%	1.33 (0.48–2.18)	1.61 (0.6–2.67)	21.1%
	Low SDI	73.9 (27.7–127.8)	189.8 (66.5–315.4)	156.8%	0.83 (0.31–1.44)	0.99 (0.34–1.64)	19.3%
	Middle SDI	1128.3 (400.3–1857.7)	3095.5 (1146.5–5214.3)	174.4%	2.98 (1.06–4.9)	3.24 (1.2–5.47)	8.7%
Beryllium	High-middle SDI	45.1 (35.3–55.5)	87 (69.6–107.8)	92.9%	0.12 (0.1–0.15)	0.12 (0.1–0.15)	0
	High SDI	17.9 (14.7–21.3)	27.2 (22.7–32)	52%	0.05 (0.04–0.06)	0.04 (0.04–0.05)	−20%
	Low-middle SDI	14.7 (11.5–18.6)	38.6 (31–47.6)	162.6%	0.07 (0.05–0.08)	0.08 (0.06–0.09)	14.3%
	Low SDI	3.9 (2.8–5.2)	9.4 (7.3–12)	141%	0.04 (0.03–0.06)	0.05 (0.04–0.06)	25%
	Middle SDI	53 (42.2–64.2)	138.2 (109.9–171.3)	160.8%	0.14 (0.11–0.17)	0.14 (0.12–0.18)	0
Cadmium	High-middle SDI	116.9 (93.9–142.5)	232.9 (187.5–283.9)	99.2%	0.32 (0.26–0.38)	0.32 (0.26–0.38)	0
	High SDI	50.7 (42.3–59.1)	70.3 (59.3–81.9)	38.7%	0.15 (0.12–0.17)	0.11 (0.09–0.13)	−26.7%
	Low-middle SDI	29.5 (23.7–36.6)	80.9 (66.1–95.6)	174.2%	0.13 (0.11–0.16)	0.16 (0.13–0.19)	23.1%
	Low SDI	7.4 (5.5–10.2)	19.2 (14.8–24.7)	159.5%	0.08 (0.06–0.11)	0.1 (0.08–0.13)	25%
	Middle SDI	111.8 (91.2–134.2)	307.9 (247.4–375.1)	175.4%	0.3 (0.24–0.36)	0.32 (0.26–0.39)	6.7%
Chromium	High-middle SDI	238.5 (204.1–277)	492.9 (412.5–593.9)	106.7%	0.64 (0.55–0.75)	0.67 (0.56–0.8)	4.7%
	High SDI	113.4 (101.3–127)	163 (145.3–182.7)	43.7%	0.32 (0.29–0.36)	0.25 (0.23–0.28)	−21.9%
	Low-middle SDI	56.7 (47.7–67.1)	166.8 (141.7–191.3)	194.2%	0.26 (0.21–0.3)	0.33 (0.28–0.38)	26.9%
	Low SDI	14.5 (11–19.3)	39.6 (32–48.2)	173.1%	0.16 (0.12–0.22)	0.21 (0.17–0.25)	31.2%
	Middle SDI	216.6 (186.6–250.2)	636.3 (529.5–760.3)	193.8%	0.57 (0.49–0.66)	0.67 (0.56–0.79)	17.5%
DEE	High-middle SDI	2750.6 (2300.8–3232.3)	5675.9 (4775–6742)	106.4%	7.44 (6.23–8.74)	7.73 (6.49–9.22)	3.9%
	High SDI	1377.9 (1207.5–1564.4)	2173.4 (1917–2478.6)	57.7%	3.93 (3.48–4.43)	3.39 (3–3.85)	−13.7%
	Low-middle SDI	814.2 (680.5–970.2)	2569.5 (2203.3–3023.4)	215.6%	3.66 (3.06–4.36)	5.15 (4.44–6.06)	40.7%
	Low SDI	232.1 (174.4–315)	648.6 (518.8–802.3)	179.4%	2.61 (1.96–3.56)	3.36 (2.67–4.18)	28.7%
	Middle SDI	2857.4 (2405.8–3361.1)	8636.9 (7234.9–10261.1)	202.3%	7.54 (6.37–8.86)	9.08 (7.64–10.76)	20.4%
Nickel	High-middle SDI	1916.7 (−112.1–5558.9)	2931.6 (223.5–7502.2)	53%	5.16 (−0.29–14.91)	3.97 (0.31–10.11)	−23.1%
	High SDI	1597.4 (−667–5933.8)	2016.5 (−810.2–7336.3)	26.2%	4.54 (−1.89–16.89)	3.06 (−1.2–11.11)	−32.6%
	Low-middle SDI	352.3 (72–788.8)	911.2 (185.2–2032.4)	158.6%	1.59 (0.33–3.55)	1.83 (0.37–4.09)	15.1%
	Low SDI	92 (18.4–208.5)	223.2 (42–511.8)	142.6%	1.03 (0.21–2.34)	1.16 (0.22–2.66)	12.6%
	Middle SDI	1287.6 (265.8–2833.2)	3243.2 (669.9–7197)	151.9%	3.4 (0.7–7.5)	3.4 (0.7–7.54)	0
PAHs	High-middle SDI	830.1 (682.4–999.1)	1710.1 (1383.4–2053.3)	106%	2.24 (1.84–2.7)	2.33 (1.89–2.8)	4%
	High SDI	397.8 (339.1–460.5)	584.7 (500.4–669)	47%	1.14 (0.96–1.32)	0.91 (0.78–1.04)	−20.2%
	Low-middle SDI	204.6 (165.5–252)	599.2 (492.2–722.1)	192.9%	0.92 (0.75–1.13)	1.2 (0.99–1.45)	30.4%
	Low SDI	53.1 (39.7–72.5)	144 (111.9–183.5)	171.2%	0.6 (0.44–0.82)	0.75 (0.58–0.96)	25%
	Middle SDI	764.9 (631.2–908.4)	2232.6 (1798.2–2683.4)	191.9%	2.02 (1.67–2.4)	2.34 (1.87–2.81)	15.8%
Silica	High-middle SDI	11436.9 (4652.5–18215.6)	16316 (7153.2–26016.7)	42.7%	30.75 (12.56–48.95)	22.06 (9.68–35.14)	−28.3%
	High SDI	9688.6 (2533–16608.2)	12308.5 (3255–20674.7)	27%	27.5 (7.24–46.86)	18.55 (5–31.3)	−32.5%
	Low-middle SDI	2051.4 (912.1–3253.4)	5526.7 (2529.5–8663)	169.4%	9.22 (4.11–14.59)	11.08 (5.06–17.31)	20.2%
	Low SDI	559.5 (241.1–928.8)	1413.9 (614.4–2278.1)	152.7%	6.28 (2.71–10.4)	7.31 (3.19–11.77)	16.4%
	Middle SDI	6920.5 (3210.4–10971.8)	17394.3 (7754.4–27650.8)	151.3%	18.26 (8.48–28.88)	18.24 (8.16–28.88)	−0.1%

*OCs, occupational carcinogens; DALY, disability adjusted life year; ASDR, age-standardized DALY rates; SDI, social demographic index; TBL cancer, tracheal, bronchus and lung cancer; DEE, diesel engine exhaust; PAHs, polycyclic aromatic hydrocarbons; UI, uncertainty interval*.

### Joinpoint Analysis

**Figure 3:**
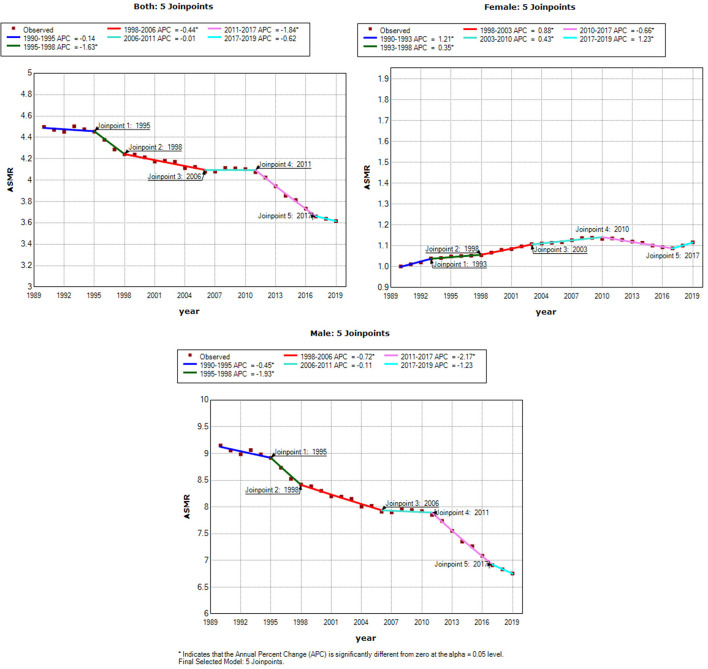
Joinpoint analysis of trend in age-standardized mortality rates (ASMR) of tracheal, bronchus and lung cancer exposure to occupational carcinogens. APC, the annual percentage change.

**Figure 4:**
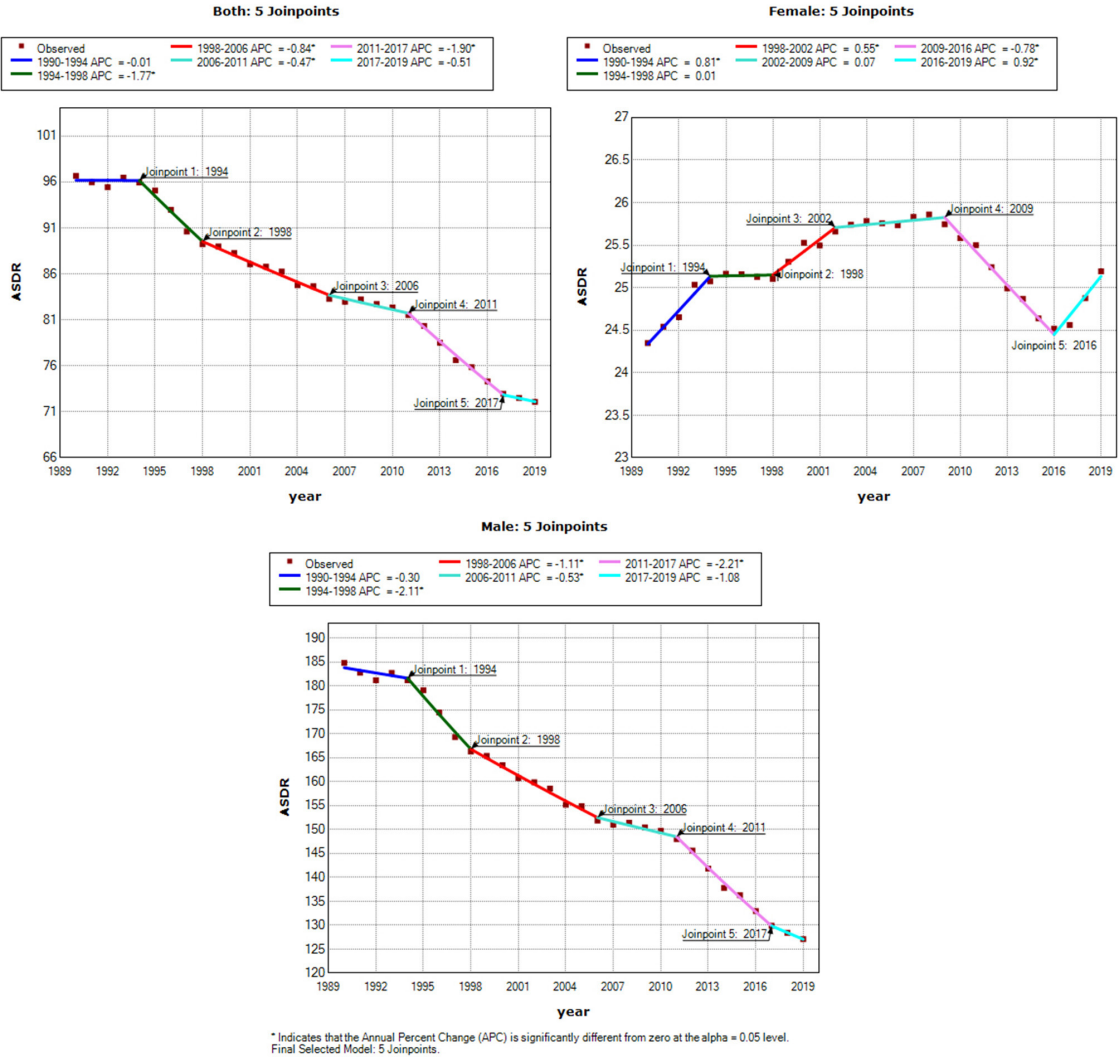
Joinpoint analysis of trend in age-standardized DALYs rates (ASDR) of tracheal, bronchus and lung cancer exposure to occupational carcinogens. APC, the annual percentage change.

Figures 3, 4 show the results of the Joinpoint analysis of trends in ASMR and ASDR of OC-attributable TBL cancer between 1990 and 2019. The Joinpoint regression results show that there were five trends in ASMR and ASDR, with a downward trend in both sexes and in men in ASMR and ASDR, respectively. The fluctuations in ASMR were small in women, although a slow increase was observed between 2017 and 2019, with overall AAPC values of 0.4 (0.3–0.4). However, the ASDR increased from 1990 to 2009, decreased from 2009 to 2016, and then increased from 2016 to 2019, with overall AAPC values of 0.1 (0–0.2) (Table 3).

**Table 3:** Trends in age-standardized mortality rates (ASMR) and age-standardized DALYs rates (ASDR) of TBL cancer, 1990–2019.

**Classify**		**Both**	**Female**	**Male**
ASMR	AAPC (95% CI)	−0.7^*^ (−0.9, −0.6)	0.4^*^ (0.3, 0.4)	−1.0^*^ (−1.2, −0.8)
	*P*-value	<0.001	<0.001	<0.001
ASDR	AAPC (95% CI)	−1.0^*^ (−1.1, −0.8)	0.1^*^ (0–0.2)	−1.3^*^ (−1.4, −1.1)
	*P*-value	<0.001	0.008	<0.001

*APC, annual percentage change; AAPC, average annual percentage change; CI, confidence interval; TBL cancer, tracheal, bronchus and lung cancer*.

* *p < 0.05*.

## Discussion

Many carcinogens are associated with a substantial disease burden at the global, regional, and national levels, and occupational diseases are attracting increasing attention (7, 9). The majority of occupational-associated malignancies are lung cancers (10). TBL cancers are primarily a consequence of population growth and aging, and they impose an enormous burden on society worldwide. It is reported that although global ASMR and ASDR are decreasing, Several countries were found to have increasing trends (7). The present results confirm this view, showing that the ASMR and ASDR of TBL cancer related to exposure to OCs decreased in both sexes and in men, respectively, from 1990 to 2019, whereas the death number increased. The increase was higher in men, as these occupations are predominantly male. Joinpoint analysis showed that the fluctuations in ASMR and ASDR were small in women; however, the ASMR and ASDR have increased in women in recent years, suggesting that this requires increased attention.

Nine OCs associated with TBL cancer were identified based on GBD 2019. Asbestos was the leading cause of death and DALYs for TBL cancer related to exposure to OCs, consistent with prior research (5). Although asbestos has been banned in more than 50 countries (11), it was not completely banned. In 2012, five countries produced 99% of the world's asbestos mines according to the Mineral Commodity Summary (12). For most developing countries, the burden of asbestos-related cancers is unknown. In this study, DEE was associated with the largest increase in deaths, whereas beryllium, cadmium, chromium, and PAHs caused a more than 2-fold increase in death number. Cadmium, chromium, DEE, and PAHs were associated with an increase in ASDR, and DEE was associated with the largest growth in ASDR. Millions of workers around the world are exposed to DEE (13). Kim et al. estimated that the percentage of lung cancers attributable to DEE exposure was 2.4% in Canada (14). DEE exposure caused 1.8% of lung cancers in the UK, making DEE the third most significant cause of occupational lung cancer burden after asbestos (15). It is important to protect miners from DEE-related TBL cancer and to continue reducing exposure to DEE. To prevent disease development, regulating industry with standardized occupational exposure limits remains a key factor (7). As many researchers have suggested, it is necessary to carry out a small-scale evaluation of the implementation of these initiatives, so as to identify cost-effective and replicable programs (7, 16).

International economic integration accelerates the free flow of production factors and shifts the risk factors of TBL cancers to lower income countries (17, 18), in particular highly polluting industries that are transferred to less developed regions. The present study showed that the increase of death number was minimal in high SDI countries, whereas low SDI, low-middle SDI, and middle SDI countries showed a significant increase compared with high SDI and high-middle SDI countries, low SDI, low-middle SDI, and middle SDI countries should pay more attention to reduce unnecessary occupational exposure. A similar trend was observed for the ASDR of TBL cancer related to exposure to OCs, with obvious increases in low SDI, low-middle SDI, and middle SDI countries. Although the ASDR is decrease in high SDI countries, but the death number is large, a series of measures such as Low-dose CT should be taken to detect TBL cancer early. TBL cancer has been linked to smoking for decades; however, the implementation of tobacco control programs and other health policies has decreased the prevalence of smoking (1, 19). Similar to the tobacco control measures, additional health policies should be proposed in low SDI, low-middle SDI, and middle SDI countries.

This study had several limitations. First, because aggregate data were obtained at the global level rather than the national level, the conclusions may not be applicable to each country. Second, possible unrecognized OC interactions between occupational and other risk factors were not considered. Third, each OCs related to TBL cancer should be further investigated.

## Conclusion

Although the ASMR and ASDR of TBL cancer related to exposure to OCs decreased in both sexes globally, attention should be paid to the recent slow increase in women. In addition, the death number increased, which may bring a heavy burden to society. Asbestos remains the biggest occupational cause of TBL cancer. Although the TBL cancer burden related to exposure to OCs decreased in high SDI countries, low SDI and low-middle SDI countries experienced varying levels of growth in TBL cancer burden related to other OCs (except asbestos).

## Data Availability Statement

Publicly available datasets were analyzed in this study. This data can be found at: https://ghdx.healthdata.org/gbd-results-tool.

## Author Contributions

HaL wrote the manuscript. ZW and JuZ conceived the study and provided guidance. JG and HoL collected and analyzed data. All authors contributed to the analysis and reviewed the manuscript.

## Conflict of Interest

The authors declare that the research was conducted in the absence of any commercial or financial relationships that could be construed as a potential conflict of interest.

## Publisher's Note

All claims expressed in this article are solely those of the authors and do not necessarily represent those of their affiliated organizations, or those of the publisher, the editors and the reviewers. Any product that may be evaluated in this article, or claim that may be made by its manufacturer, is not guaranteed or endorsed by the publisher.
